# Progress Update on STING Agonists as Vaccine Adjuvants

**DOI:** 10.3390/vaccines13040371

**Published:** 2025-03-31

**Authors:** Yanru Shen, Weijin Huang, Jianhui Nie, Li Zhang

**Affiliations:** 1 Division of HIV/AIDS and Sex-Transmitted Virus Vaccines, Institute for Biological Product Control, National Institutes for Food and Drug Control (NIFDC), Beijing 102629, China; yanru0613@sina.com (Y.S.); huangweijin@nifdc.org.cn (W.H.); niejianhui@nifdc.org.cn (J.N.); 2 WHO Collaborating Center for Standardization and Evaluation of Biologicals, Beijing 102629, China; 3 NHC Key Laboratory of Research on Quality and Standardization of Biotech Products and NMPA Key Laboratory for Quality Research and Evaluation of Biological Products, Beijing 102629, China

**Keywords:** STING, STING agonists, vaccine adjuvants, infectious diseases, cancer

## Abstract

Low antigen immunogenicity poses a significant challenge in vaccine development, often leading to inadequate immune responses and reduced vaccine efficacy. Therefore, the discovery of potent immune-enhancing adjuvants is crucial. STING (stimulator of interferon genes) agonists are a promising class of adjuvants which have been identified in various immune cells and are activated in response to DNA fragments, triggering a broad range of type-I interferon-dependent immune responses. Integrating STING agonists with vaccine components is an ideal strategy to bolster vaccine-induced immunity to infections and cancer cells. Several STING agonists are currently under investigation in preclinical studies and clinical trials; however, some have shown limited efficacy, while others exhibit off-target effects. To ensure safety, they are typically delivered with carriers that exhibit high biocompatibility and insolubility. In this review, we present the latest research on natural and synthetic STING agonists that have been effectively used in vaccine development, and summarize their application in adjuvant preventive and therapeutic vaccines. Additionally, we discuss the safety of STING agonists as vaccine adjuvants by reviewing potential delivery strategies. Overall, incorporating STING agonists into vaccine formulations represents a significant advancement in vaccine research with the potential to significantly enhance immune responses and improve vaccine efficacy. However, ongoing research is still required to identify the most effective and safe delivery strategies for STING agonists, as well as to evaluate their long-term safety and efficacy in clinical trials.

## 1. Introduction

**Figure 1:**
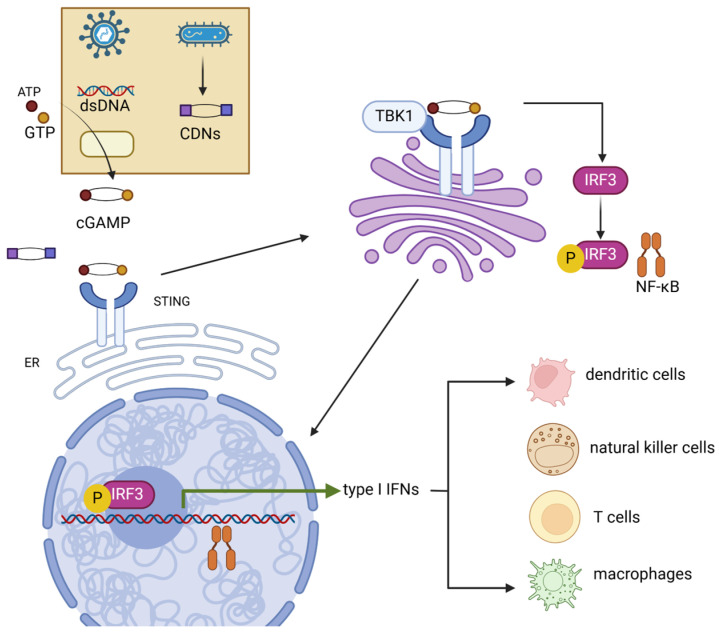
Mechanism of cGAS-STING signaling pathway. STING can be activated by both dsDNA and CDNs produced by pathogens. Activated STING transferred from the ER to the Golgi apparatus, where STING promotes the phosphorylation of IRF3 and NF-κB by TBK1. IRF3 and NF-κB then induce the production of type-I IFNs. These cytokines then promote the maturation of several immune cells such as dendritic cells, natural killer cells, T cells, and macrophages. dsDNA, double-strand DNA; CDNs, cyclic dinucleotides; TBK1, TANK-binding kinase 1; IRF3, interferon regulatory factor 3; NF-κB, nuclear factor kappa B.

The mammalian immune system contains a DNA sensor protein, stimulator of interferon genes (STING), located on the endoplasmic reticulum and Golgi apparatus. STING plays a critical role in pathogen recognition during infections and belongs to the family of cytosolic pattern recognition receptors (PRRs), which recognize and bind pathogen-associated molecular patterns (PAMPs) [1]. STING receptors specifically sense short-length cytosolic double-strand DNA (dsDNA) or cyclic dinucleotides (CDNs) produced by microorganisms [2,3], triggering downstream immunogenic responses—including both innate and adaptive immunity to infections [4]—despite the distinctive process of STING activation occurring in the event of CDN or dsDNA binding. With the presence of dsDNA, the triggering of the STING signaling pathway generally includes three steps (Figure 1). First, during the invasion of pathogens, dsDNA is detected by a cytosolic DNA sensor cyclic GMP-AMP synthase (cGAS) and binds to the nucleotidytransferase domain of cGAS [4]. Binding initiates a catalytic reaction between ATP and GTP, leading to the production of cGAMP, a CDN second messenger that is sensed by STING [5]. The activation of STING is then initiated via the binding of cGAMP [6], causing a conformational change and promoting STING oligomerization [7]. In addition to dsDNA, bacteria secrete other CDNs, such as c-di-AMP, which is akin to cGAMP and can directly bind with STING without cGAS involvement, activating downstream signaling [8]. While experimental evidence has proven that DNA and RNA virus infections trigger the STING signaling cascade [9,10], the mechanisms remain unclear. Hypothetically, the genetic material residues injected into the host cell nucleus by DNA viruses might unintentionally be detected by cGAS [11]. On the other hand, binding RNA with cGAS restricts the production of cGAMP. The activation of STING might be in response to the destructed organelles induced by RNA virus infections [12,13].

Following activation, oligomerized STING undergoes palmitoylation and translocates from the ER to the Golgi via the ER–Golgi intermediate compartment (ERGIC) [14,15]. In the Golgi, STING activates TANK-binding kinase 1 (TBK1), which phosphorylates STING [16]. This process requires the oligomerization of both STING and TBK1 proteins [17]. STING then acts as a scaffold, promoting the TBK1-dependent recruitment and phosphorylation of interferon regulatory factor 3 (IRF3) and NF-κB. This leads to the translocation of these factors into the nucleus, where they upregulate innate immune genes—typically type-I interferons (e.g., IFN-α and IFN-β)—and then induce the expression of various IFN-stimulated genes (ISGs), such as IFIT1, IFIT2 IFI6, and MX2, consequently resulting in a robust immune response [10,15,18,19,20]. STING ligand–receptor interactions occur in various immune cells within the tumor microenvironment, including dendritic cells, macrophages, T cells, and B cells, mediating the secretion of antitumor cytokines and chemokines such as IL-18 and CXCL10 [21,22,23,24]. STING and cGAS knockout mice have shown significantly decreased type-I IFN production [25,26,27]. Despite Anghelina et al. suggesting that no decrease in viral clearance capability against infection was observed in STING and cGAS knockout mice [26], deficient pathogen clearance was reported in STING knockout mice against Staphylococcus aureus and West Nile virus [25,27]. Moreover, cells without type-I IFN receptor expression are unable to be activated by the STING signal. A study by Nicolai et al. found that DCs with IFN-α receptor depletion were unable to react to STING-induced IFN-α, accompanied by the loss of downstream immune responses [28]. Accordingly, the STING signaling pathway is an essential component of mammalian immune systems.

Despite advances, the development of prophylactic vaccines against infections and therapeutic vaccines for tumors remains challenging due to issues like drug inefficacy and unpredictable side effects. As such, STING agonists have emerged as promising adjuvants in vaccine development due to their strong immunogenic properties. In this review, we discuss the role of the STING pathway in vaccine response and explore recent studies utilizing STING agonists as adjuvants for anti-infection and antitumor vaccines.

## 2. The STING Signaling Pathway Acts as the Bridge Between Innate and Adaptive Immunity

### 2.1. Innate Immunity

STING triggers type-I interferon (IFN) secretion to activate various immune cells, collectively enhancing host immunity to infection and tumors. In response to specific immune signals and stimuli, macrophages undergo a polarization process that requires STING-dependent NF-κB activation, differentiating into the pro-inflammatory M1 subtype [29,30]. STING also facilitates the activation of natural killer (NK) cells. Tumors with chromosomal instability (CIN) accumulate cytosolic dsDNA, which reacts with STING and triggers the secretion of type-I IFNs and IL-15 to establish NK cell responses [31,32,33,34]. In addition, studies have noted that the activation of STING and the type-I IFNs themselves directly promote dendritic cell (DC) responses. Type-I IFNs enhance DC maturation via upregulating ligands such as CD40, HLA-DR, CD80, and CD86 [35,36]. Those immune cells possess highlighted tumoricidal capabilities [37,38,39,40], indicating STING as a remarkable antitumor target.

Autophagy, a cellular degradation process essential for maintaining homeostasis, is also part of the innate immune response [41]. The STING signaling pathway is also involved in autophagy, in response to the pathogen invasion [41]. Viruses such as HSV-1 induce the STING signaling cascade and active LC3-dependent autophagy [42]. Peculiarly, during M. tuberculosis infection, macrophages recruit cGAS to bind to bacterial extracellular DNA (eDNA), generating cGAMPs to trigger STING activation and subsequently initiating a ubiquitin-dependent autophagy pathway [43,44]. STING-mediated autophagy also plays a role in tumor suppression. Zalcitabine, an antiviral drug reported by Li et al., is capable of generating ferroptotic autophagy against pancreatic carcinoma through DNA-stress-activated STING signaling that is triggered by excessive iron uptake [45].

### 2.2. Adaptive Immunity

The STING pathway bridges innate and adaptive immunity by regulating T-cell differentiation. As T-cell differentiation is one of the characteristic features of host adaptive immune response, endogenous STING was asserted to prohibit T-cell proliferation and promote TH1 cell differentiation via regulations of IRF3 and TFN-β [46,47]. Apart from TH1 cells, CD4+ T-cell intrinsic STING is capable of enhancing the differentiation of TH9 cells, though this requires the collaboration of the mTOR signaling pathway [46]. CD8+ T-cell differentiation is also elicited by the STING signaling pathway. Zhang et al. reported that the hepatocellular carcinoma-intrinsic STING signaling elevation enhances the antitumor CD8+ T-cell infiltration [48]. The STING pathway plays an important role in augmenting the CD8+ T-cell cross-priming process [40].

A previous study showed that host immune responses are highly deactivated during infections and in the tumor microenvironment [49,50], which might decrease the efficacy of vaccines. Formulating STING agonists with vaccines can trigger intrinsic STING-induced immune responses. These immune responses, including activation of dendritic cells, natural killer cells, macrophages, and lymphocytes, lead to the secretion of cytokines and chemokines to exterminate the infections and tumor cells. Thus, STING agonists are considered ideal adjuvants that can revive the immunosuppressed environment, significantly enhancing vaccine efficacy.

## 3. STING Agonist Selection for Vaccine Adjuvants

### 3.1. Natural Agonists

c-di-AMP and c-di-GMP: During vaccine development, efficient agonists are required to activate the STING signaling pathway to derive vast immune responses which defend against infections and cancers. The most frequently considered chemicals are natural ligands for STING. As we concluded above, STING is activated by a group of substances named CDNs that are produced by pathogens, including c-di-AMP and c-di-GMP [11,51,52]. Accordingly, CDNs have been considered vaccine adjuvants to promote immunogenicity against Klebsiella pneumoniae for the past decade [53]. Chandra et al. revealed that using c-di-GMP as a vaccine adjuvant efficiently exterminated cancer cells in vaccination targeting metastatic breast cancer [54]. In addition, the subunit vaccine formulated with the 6 kDa early secretory antigenic target (ESAT-6) and co-administered with c-di-AMP triggered lung innate lymphoid cell response and successfully eliminated M. tuberculosis infections, rather than activating CD8^+^ T-cell differentiation [55].

cGAMP: As shown above, the interaction between cGAS and dsDNA leads to the synthesis of cGAMP, which is also a type of CDN and acts as the direct ligand of STING [56]. cGAMPs are naturally present in various types of isoforms, where the endogenous cGAMP in mammalian cells contains an identical 3′-5′ phosphodiester linkage, named 2′3′-cGMAP. This linkage was previously suggested to possess a higher binding affinity to STING than other isomers of cGAMP, such as 3′3′-cGAMP encoded by Vibrio Cholera that functions in bacterial colonization [57]. On the contrary, the use of 2′3′-cGAMP and 3′3′-cGAMP as vaccine adjuvants in other studies promoted immunity to Bacillus anthracis-derived anthrax and demonstrated an equivalent response of both types of cGAMP in stimulating mucosal immunity [58]. The adjuvant capability of cGAMPs as STING activators requires further investigation.

dsDNA: Notably, although dsDNA is the major ligand of cGAS, few studies have demonstrated its adjuvant capability. Miura and colleagues first discovered that plasmid DNA (pDNA) coated with their synthesized nanocarrier KALA-MEND induced a STING-dependent antiviral response, with the downstream activation of T-cells and DCs [59]. Similarly, pDNA-based agonists are also reported to be efficient in tumor treatment. Huang et al. combined pDNA, which also acts as a STING activator, with immunosuppressive-factor-inhibiting small interfering RNA (siRNA) in nanoparticles (NPs), which adequately eliminated hepatocellular carcinoma [60]. Although STING has been considered a DNA sensor, it can also be activated via a DNA/RNA hybrid [61]. These findings suggest a potential candidate for vaccine adjuvant that targets STING signaling pathways.

Astaxanthin (ASTA): In addition to commonly used agonists, Li and colleagues have recently discovered that astaxanthin, a natural compound produced by marine organisms, is a potent agonist of STING. In their study, ASTA alleviated the lipid-peroxidation-derived STING carbonylation, which suppresses the translocation of STING. This inhibition promotes the activation of STING and leads to the upregulation of type-I IFNs and ISGs. However, no direct binding between ASTA and STING has been reported, indicating that ASTA might be an indirect agonist. Specifically, ASTA showed a rescue effect on HSV-1-induced oxidative stress, further eliciting the STING pathway to reduce viral replication [62]. The study highlighted that ASTA has a high potential as an antivirus vaccine adjuvant.

### 3.2. Synthetic Agonists

DMXAA: Despite natural STING agonists being able to directly trigger the STING signaling pathway, they are usually characterized by unstable, synthetic chemicals and thus must be developed to achieve higher treatment efficiency. The antitumor drug 5,6-dimethylxanthenone-4-acetic acid (DMXAA) was earlier discovered to reduce tumor blood flow, hence leading to hemorrhagic tumor necrosis [63], and it was later reported that it can be used as a potent vaccine adjuvant that could trigger an antitumor effect [64,65]. Subsequent studies confirmed that DMXAA facilitates immune responses by interacting with STING and can also be used to augment antiviral vaccines against vesicular stomatitis virus and influenza [66,67]. However, human-encoded STING (hSTING) was unable to interact with DMXAA and, thus, an adjuvant study was not successful in human cells [68]. To solve this, Gao et al. created an engineered hSTING protein, showing two amino acids on the DMXAA binding pocket, and the lid of the pocket is responsible for DMXAA binding [69]. Further study investigating a DMXAA analogue compound that can act as the ligand for hSTING is required.

ADU-S100: To achieve interactions between synthetic drugs and hSTING, various modified CDNs that enable the activation of hSTING were developed [70]. Corrales et al. created an intertumoral-administration synthetic CDN compound, named ML-RR-S2-CDA/ADU-S100, which binds to both hSTING and murine STING, generating antitumor effects via T-cell response [70,71,72]. ADU-S100 was under study in a clinical trial; however, all studies have been terminated, two of which were due to a lack of substantial antitumor activity (Table 1).

MK-1454: Another intertumoral hSTING agonist, MK-1454, is also undergoing clinical trials. Despite this agonist being suggested to harbor a boosting effect when co-administered with humanized anti-PD-1 antibody, adverse side effects were still observed, indicating a lack of efficiency [70,73,74]. However, due to the difficulties in the accessibility of intertumoral injection to solid tumors, studies were also focused on systemic administration.

diABZI: Ramajulu and colleagues linked two amidobenzimidazoles (ABZIs) to create a synergetic STING agonist, diABZI. This compound allows injection via the intravenous route that agitates sustained immune response [72,75]. The following study showed that diABZI compounds bind to both murine and human STING, generating outstanding antiviral and antitumor effects against parainfluenza, human rhinovirus, and SARS-CoV-2 and facilitating STING-induced brain tumor regression [76,77,78]. However, treatment using even a low dose of diABZIs as STING stimulators might lead to unexpected acute respiratory distress syndrome, highlighting a safety concern of diABZIs [79].

Other synthetic compounds: Akin to DMXAA, early developed antivirus STING agonist CMA was observed to elicit robust TBK1/IRF3 activity, despite being unable to interact with hSTING [80]. CMA first highlighted the potential of artificial-compound-derived STING cascade activation in antiviral drugs. Subsequently, Sali et al. presented another chemical substance, G10, inducing STING-dependent antivirus factor type-I and type-III IFN, whereas the data demonstrated that G10 might indirectly bind to hSTING [81]. Nonetheless, the direct interaction between G10 and hSTING has recently been proved, although in a low-affinity binding manner [82], indicating that using G10 as a STING-related adjuvant might be insufficient compared to other chemicals with strong interactions. A study by Hou et al. examined the capacity for triggering the STING signaling pathway with six synthesized acridone analogues (2g, 9g, 12b, 1b, 1c, and 12c) and demonstrated that chemical 12b had a higher activity on elicit type-I IFNs and directly bound to both murine and human STING. Henceforth, 12b was considered an efficient compound with significant potential as an adjuvant for both antiviral and antitumor vaccines [83].

**Table 1:** STING agonists that are currently in clinical trials [84,85,86,87,88,89,90,91,92,93,94,95,96,97,98,99,100,101,102,103,104].

Drug	Molecular Types	Conditions	Route	Combination	Phase	Status	Gov ID
ADU-S100 (MIW815)	CDN	Solid tumors/lymphoma	IV	Anti-CTLA4 mAb	I	Terminated	NCT03172936
		HNSCC	IT	PD-L1 mAb	II	Terminated	NCT03937141
		Advanced/metastatic solid tumors/lymphoma	IV	Ipilimumab	I	Terminated	NCT02675439
Ulevostinag (MK-1454)	CDN	Solid tumors/lymphoma	IT	Pembrolizumab (MK-3475)	I	Completed	NCT03010176
		HNSCC	IT	Pembrolizumab	II	Completed	NCT04220866
CRD3874-SI	Small-molecule	Acute myeloid leukemia	n.s.	Monotherapy	I	Recruiting	NCT06626633
GSK3745417	Non-CDN	Neoplasms	IV	Dostarlimab	I	Active, not recruiting	NCT03843359
		Acute myeloid leukemia	IV	Monotherapy	I	Terminated	NCT05424380
SYNB1891	Engineered bacteria CDN carrier	Metastatic solid neoplasm/lymphoma	IT	Atezolizumab	I	Terminated	NCT04167137
TAK-676 (Dazostinag)	CDN	HNSCC/TNBC/NSCLC	IV	Pembrolizumab/radiation therapy	I	Completed	NCT04879849
		Solid neoplasm	IV	Pembrolizumab/platinum/5-fluorouracil	I/II	Recruiting	NCT04420884
IMSA-101	CDN	Adult solid tumors	IT	ICI/IO	I/II	Completed	NCT04020185
		Adult solid tumor (Rollover study)	IT	ICI	I	Active, not recruiting	NCT06026254
		mRCC/oligoprogressive metastatic disease	IT	Monotherapy	II	Not yet recruiting	NCT06601296
SNX281	N/A	Advanced solid tumor/lymphoma	IV	Pembrolizumab	I	Terminated	NCT04609579
CDK-002 (exoSTING)	CDN carrier	Advanced solid tumors	IT	Monotherapy	I/II	Completed	NCT04592484
ONM-501	CDN	Advanced solid tumor/lymphoma	IT	Cemiplimab	I	Recruiting	NCT06022029
BI 1387446	CDN	Neoplasms	IT	Ezabenlimab (BI 754091)	I	Completed	NCT04147234
TAK-500	CDN	Metastatic solid tumors	IV	Pembrolizumab	I/II	Recruiting	NCT05070247
BI 1703880	Small-molecule	Solid tumors	IV	Ezabenlimab	I	Recruiting	NCT05471856
SB 11285	Small-molecule	Advanced solid tumors	IV	Atezolizumab	I	Completed	NCT04096638
XMT-2056	ADC	Solid tumors	IV	Monotherapy	I	Recruiting	NCT05514717
BMS-986301	CDN	Advanced solid tumors	IT	Nivolumab/Ipilimumab	I	Completed	NCT03956680

HNSCC, head and neck squamous cell carcinoma; TNBC, triple-negative breast cancer; NSCLC, non-small cell lung cancer; mRCC, metastasis renal carcinoma; IV, intravenous; IT, intratumoral; n.s., non-specified.

## 4. Delivery of STING Agonists

### 4.1. Organic Particles

Several STING agonists, such as cGAMP-type compounds, are biodegradable by enzymes or water-soluble. Liposomal nanoparticles (LNPs) are among the most widely used agonist carriers to prevent off-target drug effects and undesired systemic inflammation. Examples of LNPs for vaccines against cancer have already been shown to be effective against breast cancer [105], melanoma [106], and lung cancer [107]. At present, a system named NanoSTING, a novel development for anti-infection vaccines developed by Sefat et al., can be mixed with different types of antigens on the surface of the particles. This formula has been intranasally immunized to BALB/c mice and has led to prolonged protection time and enhanced STING-dependent cellular immunity to Mtb, SARS-CoV-2, and influenza [108]. Similarly, another study encapsulated cGAMP with pulmonary surfactant (PS) constituent-based LNPs, allowing adjuvant transport into the STING-presenting cells through the PS layers. This combination elicits strong CD8+ activation to defend against H1N1 infection and is capable of generating cross-protection against other types of influenza over 6 months [109].

Natural CDNs are considered low bioavailability due to their negative charges and hydrophilic molecule properties [110]. Due to the controllable drug release of biodegradable mesoporous silica nanoparticles (MSNs), the research group of Park et al. developed a low-cost, inorganic drug delivery method using these particles to improve the STING agonist delivery system [111]. Compared to the conventional MSNs, this novel NP possesses a rapid biodegradation rate, allowing higher efficiency in agonist import [112]. With the combination of c-di-AMP, particles enable the release of the STING agonist regularly with a 1 h interval and demonstrated impressive capability to rescue mice from melanoma in vivo [111]. Recently, studies discovered that the synergic delivery of metal ions with MSNs improves the MSN-STING delivery system. Both attempts on Fe^2+^ and Mn^2+^ showed remarkable results in suppressing virus infection and tumor growth [78,113,114]. Based on the metal ion MSN configuration, Li et al. loaded Mn^2+^ MSNs with antitumor drug CDDP and a non-CDN STING agonist SR-717. The results demonstrated prolonged drug effects and tumor-specific accumulation, which sufficiently revived the immunosuppression tumor microenvironment (TME) [115]. Another study by Dou et al. found that using Mn^2+^ MSNs as delivery cargo for the DP-1 inhibitor metformin can increase the drug concentration in the TME and activate STING to intensify local immune responses [116].

### 4.2. Inorganic Particles

Despite the effectiveness of organic NPs, their complex synthesis procedures are considered inappropriate for large-scale vaccine production. As metal cations have been found to play significant roles in STING activation [117,118], metal-based nanodelivery systems, with their simple assembly processes, have been introduced. Liu et al. developed a STING-activating, Mn-based, layered, double-hydroxide nanoadjuvant with the intention of coupling the glutathione depletion response and STING signaling pathway for DC maturation. This particle is not required to combine with any adjuvant STING agonist, but based on the Mn^2+^ stimulation activity on STING itself, its combination with the model antigen ovalbumin (OVA) demonstrates superior STING-dependent innate immunity to B16-OVA melanoma [119]. In addition, the Mn^2+^ ion was found to be capable of transferring the specific macro-scale crystal of Zn^2+^ with CDNs into the particle complex Zn-Mn-CDN (ZMCP). Researchers state that this complex exhibited excellent stability and biocompatibility, effectively inducing vast cellular and antibody responses to both tumor and SARS-CoV-2 infection [120]. Luo et al. synthesized 16 lanthanide (europium) ions with cGAMP to generate a Eu-based nanoparticle, which is used in the delivery of adjuvants. This system possesses properties that include high thermal and chemical stability and simple administration that enable the derivation of the robust cellular and humoral response to B16-OVA melanoma [121].

### 4.3. Biological Particles

Compared to other types of delivery cargo, biological particles possess high biocompatibility. In one instance, cGAMP was applied via monoaxial electrospraying onto biopolymer-based acetalated dextran microparticles (Ace-DEX MPs), which degraded in the acidic endosomal environment to release the adjuvants. Formulating Ace-DEX MPs with HA influenza subunits elicited robust T-cell responses and demonstrated a 100-fold enhancement of IgG production against influenza compared to uncoated cGAMP [122]. Using iron-based biological particles, several studies have recently attempted to eliminate cancer cells by triggering tumor cell ferroptosis with a STING agonist adjuvant as an immunostimulant. Sun et al. engineered the outer membrane vesicle (OMV) from negative bacteria into NPs and carried out absorption with ferrous ions and STING agonist 4. The in vivo immunization of this NP demonstrated excellent tumor targeting and cancericidal capabilities through downregulating several antioxidative and anti-ferroptosis proteins and STING-induced CD8+ T-cell response [123]. Remarkably, Wang and colleagues engineered human ferritin into NPs as the protein is naturally capable of traveling through the blood–brain barrier. This engineered NP was modified with glioma-targeting motif and the STING agonist SR717 and was hence able to penetrate the tumor tissue of glioma. The intravenous administration of engineered ferritin in mice successfully activated numerous innate and adaptive immune cells, including T cells and NK cells, promoting tumor penetration by lymphocytes. The treatment attenuated the development of glioma and led to an 83% survival rate in a mouse model [124].

### 4.4. Biomimetic Particles

Despite high efficiency and good biocompatibility, particle-based delivery cargo faces limitations such as easily becoming stuck in the tumor vessels and being unable to reach tumor cells [125]. Bacteria- and cell-based particles were thus developed to overcome the high immunogenicity and off-target drug effects. An engineered *E. coli* strain, SYNB1891, was designed as a c-di-AMP self-producing carrier with the aim of precisely delivering agonists toward intratumor APCs, thus preventing undesired apoptosis generated by off-target agonists [126,127]. The engineering includes the insertion of a c-di-AMP-producing enzyme gene *dacA* (diadenylate cyclase), the removal of *thyA* (thymidylate synthase) that ensures the biocontainment of the bacteria and the deletion of all antibiotic resistance genes [127]. SYNB1891 not only induces innate immunity derived via the STING signaling pathway; the bacteria itself are also responsible for the activation of other PRRs. This *E. coli*-based cargo has been tested in a phase I clinical trial and has proven tolerance and efficiency [93].

Cytopharmaceutical technology has been used to develop novel drug delivery systems in recent decades. Cell-based cargos are loaded with drugs and chemical compounds ex vivo, which enables the effectiveness of drugs to be coupled with the endogenous advantages of cells to achieve superior biocompatibility, avoid off-target drug effects and prolong protection [128,129]. According to the tumor-penetrating ability of neutrophils (NEs), Hao et al. developed cytopharmaceutical NEs containing STING agonists attached to the cargo surface. The STING agonist is coated with negative hyaluronic acid-maleimide (HA-Mal) that dissolves once the NEs reach the tumor site and release the agonist for immune activation. This cytopharmaceutical NE cargo has elevated and multitudinous cellular immunity within the TME and eases the immunologically “cold” condition [128].

Virus-mimicking particles have been introduced in vaccine development as immunostimulants. Recently, Wang et al. designed a virus-like particle by representing vesicular stomatitis virus glycoprotein and calreticulin on the surfaces of engineered tumor cell-derived extracellular particles carrying cGAMP. The virus-mimicking particle has a high affinity to DCs and allows membrane fusion for the delivery of cGAMP to trigger the STING signaling pathway and promote the T-cell cross-priming. Their research regained robust T-cell infiltration response in the T41 breast cancer and B16 melanoma models [130].

## 5. Application of STING Agonists in Anti-Infection Vaccines

### 5.1. Vaccines Against Bacterial Infection

STING agonists are emerging adjuvants for vaccines against infectious diseases. Dis et al. first designed a novel subunit vaccine by combining a STING agonist cyclic-dyguanylate (CDG) with the fusion protein of Mtb antigens to compensate for the lack of immune activation of Bacille Calmette–Guérin (BCG) vaccine. The co-administration led to a more than 20-fold increase in antigen-specific T-cell response and prolonged protection targeting Mtb compared to BCG [131,132]. Following the widespread adoption of organic delivery platforms, a recent study by Howlett et al. loaded cGAMP and Mtb antigens into metal-modified organic particles ZIF to attain higher vaccine efficiency. The inserted Zn^2+^ and Mn^2+^ ions further boosted STING-dependent cellular responses to Mtb [133].

### 5.2. Influenza

Developed vaccines against viruses (e.g., influenza) are facing the problems of rapid antigen shift and drift, which may render existing vaccines obsolete [134]; thus, it is essential to investigate appropriate adjuvants that promote long-lasting protection. A cGAMP-adjuvated H5N1 vaccine attained 40-week immunization and elicited a 1000-fold increase in IgG2c production compared to administrating the vaccine alone [135]. The use of cGAMP as an adjuvant for the H7N9 vaccine also achieves 10 times the dose-sparing capability compared to administering the vaccine alone [136], indicating that STING agonists were conductive to preserving vaccine supplies and relieving financial strains during the pandemic [137].

### 5.3. SARS-CoV-2

In recent years, the COVID-19 pandemic prompted intensive antiviral vaccine development, and messenger RNA (mRNA) vaccines have become a research hotspot. Zhang et al. combined STING-agonist-derived amino lipids (SALs) together with 2-dioleoyl-sn-glycero-3-phosphoethanolamine (DOPE), cholesterol, and DMG-PEG2000 to form a lipid nanoparticle (LNP) that assists the delivery of mRNA from the SARS-CoV-2 spike protein. The intact nanoparticle system (named the SAL-LNP system) allows the SALs to bind to STING, thus eliciting a robust STING-dependent immune response to SARS-CoV-2 infection [138]. Noticeably, the preferable injection time point of the STING agonist SARS-CoV-2 vaccine is the early stage of infection. Recent studies have reported that the endogenous STING signaling pathway was enhanced during the second stage of SARS-CoV-2 invasion as the infection brings about an interaction between cytosolic self-destructed DNA that activates the STING signaling pathway, consequently leading to severe inflammatory responses in patients [139,140] and revealing that the belated administration of STING agonists during the late infection stage of SARS-CoV-2 might lead to adverse prognosis. This SARS-CoV-2-stimulated delay in innate immunity was further supported by another study. Li et al. found that the secretions of both type-I and type-III IFNs were increased at the late infection time point. To avoid undesired inflammation, they administered treatment with synthetic diABZI STING agonists at the early stage of infection and sufficiently slowed the SARS-CoV-2 invasion in mice [141], suggesting that STING agonist adjuvant vaccines should be used as early treatment to obtain the most effective protection against SARS-CoV-2. Later, Lei et al. improved this diABZI-adjuvated vaccine by combining the agonist and receptor binding domain (RBD) of the SARS-CoV-2 spike protein with a yeast-extracted β-glucan particle (GP) system named the GP-diABZI-RBD delivery system. The research group intraperitoneally injected GP-diABZI-RBD into BALB/c mice. The vaccine activated STING for extensive T-cell and humoral immunity and increased the production of IgG, neutralization antibody (nAb), and a multitude of antiviral cytokines such as IL-6, IL-10, and IL-13. This formula allows drugs to be imported into cells via phagocytosis and can generate long-lasting immunization [142].

### 5.4. HIV

Researchers combined STING agonists and HIV viral proteins for vaccine development early in the study of the STING signaling pathway. Bosch and coworkers combined the STING agonist c-di-GMP with HIV Env protein and HIV pseudovirions (Env-PVs) in a mouse model, via a subcutaneous route. However, this attempt was unable to elicit a strong humoral response [143]. Later, Hanson et al. claimed that CDNs would more effectively function when co-administered with weak immunogenic antigens. Thus, they combined HIV peptide antigen gp41 with NPs coated with c-di-GMP, which is capable of eliciting robust immune responses (CD4+ and B cells) in a murine model [144]. In addition, co-administered NPs coated with 2′3′-cGAMP with HIV1 Gag p24 sufficiently generated Gag-specific strong T-cell response and protected CB6F1 mice from HIV-1 Gag-expressing vaccinia virus [145]. Since DCs are considered critical targets for eliciting immune response to HIV-1 infection, Calvet-Mirabent et al. activated intrinsic TBK1 in DCs via co-administered poly I:C and 2′3′-di-cAM(PS)2 in hBLT mice, which promoted the maturation of DCs, further triggering polyfunctional CD4+ T-cell response. This vaccine also utilizes HIV-1 Gag peptide as an antigen, hence significantly suppressing the spread of HIV infection from the spleen to the lymph node [146]. During cell invasion, HIV develops an inositol hexakisphosphate-containing viral capsid, resulting in immune escape from STING-mediated DNA sensing [147]. Using STING agonists as HIV vaccine adjuvants, it is possible to reactivate the STING signaling pathway suppressed by HIV infection.

### 5.5. HSV

HSV infection targets the neuron cells and leads to fatal herpes simplex encephalitis (HSE) [148]. STING agonists have been demonstrated to assist in alleviating HSV-induced neuron damage. Mice intraperitoneally (i.p) injected with the synthetic agonist DMXAA were reported to have reduced viral load in the central nervous system, with an increase in survival rate of 30%. These positive effects were not observed in STING- or IRF3-deficient mice, indicating that despite relatively low efficiency, targeting the STING signaling pathway presumably has some potential against HSV-1 infection [149]. However, the study reported that the DMXAA-treated mice generated a unilateral loss of motor function, and due to safety considerations, further examination using DMXAA against HSV infection is required. To date, there is still limited research on co-administering STING agonists in vaccines against HSV.

### 5.6. Hepatitis B

STING signaling-derived IFN-β-mediated immune response is required for rescuing immunogenic weakness caused by chronic hepatitis B virus (HBV) infection. Researchers have also focused on the STING signaling pathway to discover sufficient treatment methods for this infection. Despite a study previously claiming that HBV infection plays an attenuating role in STING activation [150], stimulating STING with agonists was also proved to impair the progression of HBV infection. For instance, the treatment of DMXAA effectively repressed the replication of HBV both in vitro and in vivo by eliciting a robust immune response in macrophages through the STING cascade, causing the destruction of the cytoplasmic viral nucleocapsid [151]. Likewise, Li et al. studied the impact of STING-derived immune response on HBV infection by intraperitoneally injecting DMXAA in an Alb-Cre Tg mouse model. The application of DMXAA activated the Kupffer cells instead of hepatomas; successfully inhibited recombinant covalently closed circular DNA (rcccDNA)-derived HBC replication, hence reducing viral DNA load in mice; and eased chronic HBV-related liver fibrosis [152]. However, a study by Guo et al. claimed that no cytotoxicity effects were generated by DMXAA in hepatocyte cell cultures but also reported that DMXAA-treated NOD/SCID mice exhibited a weight loss of 8%, demonstrating a possible side effect caused by the in vivo administration of DMXAA [151]. Furthermore, Polidarova et al. have identified three CDNs, a natural STING agonist 2′,3′-cGAMP, a synthetic cGAMP analogue 3′,3′-c-di(2′F,2′dAMP) and its prodrug named bis(pivaloyloxymethyl). These CDNs are latent and efficient for controlling HBV infection. Their study compared the anti-HBV capabilities between Toll-like receptor 7 (TLR7) and STING agonists. The results proved that, rather than predominant secretion of IFNα as TLR7, STING agonists elicited other various types of anti-HBV cytokines, including TNFα, IL6, and IL1β, and had an exceptional anti-HBV effect compared to TLR7 [153]. However, the activation of STING in target cells in the study was only performed in vitro, and the efficiency of using these STING agonists against HBV requires further elucidation.

### 5.7. Anti-Parasite Vaccines

The role of STING agonists in evoking host anti-parasitic immunity has also been explored in recent years; an early attempt focused on the improvement of *Trypanosoma cruzi* vaccines [154,155]. Alberti and colleagues formulated c-di-AMP with an engineered chimeric antigen Traspain consisting of three *T. cruzi* proteins. The combination induced substantial IgG production and elicited antigen-specific CD4+ and CD8+ T-cell responses to ease the parasite-induced tissue damage [154], and the vaccine performance was further confirmed in a later study using DNA prime/boost protocols [155]. In another example, Quintana et al. produced a *T. cruiz* antigen trans-sialidase (TS) using *Lactococcus lactis* [156], then formulated the compound with c-di-AMP to construct an intranasally administered *T. cruiz* vaccine [157]. Following the successful enhancement of immune responses, this group improved the vaccine by combining two TS fragments, TSnt and TSct, with c-di-AMP [158]. The amelioration triggered prominent cytokine production at the nasopharynx-associated lymphoid tissue and systemic immune responses. Furthermore, a recent study also found that the activation of STING by cGAMP triggers a CD4+-mediated type-I regulatory CD4+ T-cell response [159]. This type of immune cell is vital in eliminating Plasmodium falciparum from causing malaria, indicating that STING agonists are adjuvants with high potential against *P. falciparum* infection.

### 5.8. STING Agonist Adjuvant Vaccines Induce Humoral Immunity to Infection

Although there is a lack of evidence supporting that endogenous STING signaling positively regulates humoral immunity during infection, vaccine-induced STING activation was reported to promote humoral immune response, including B cell activation and antibody secretion. Zhong et al. found that the blockage of type-I IFN receptor after STING-stimulating poxviral vector vaccination strongly impeded B cell activities and led to a deficient antibody generation capability in mice [160]. The protection offered by existing vaccines against SARS-CoV-2 rapidly decreases after a second dose [161]; hence, Liu et al. designed the synthesized STING agonist CF501 and reported an impressive antibody persistence after mouse immunization. Researchers combined CF501 and Fc fragments from human IgG with the receptor binding domain (RBD) from SARS-CoV-2 variants, which induced potent and long-lasting RBD-specific IgG production against SARS-CoV-2 virus in mice, rabbits, and rhesus macaques [162]. Importantly, Liu and colleagues also compared the humoral response generated by alum and c-di-AMP. Despite being slightly diminished compared to CF501, the IgG level significantly increased with the adjuvant for c-di-AMP compared to the vaccine with alum only [162]. On the contrary, another study by Landi et al. reported a similar level of humoral response induced by alum and c-di-AMP, and the c-di-AMP was particularly efficient in exerting CD4+ T-cell response [163]. Similarly, the STING signaling pathway was also asserted to play a modest role in humoral response but trigger early cellular immunity when activated by adenovirus DNA [27], indicating that STING-induced humoral responses are dependent on agonists. However, most studies of STING agonist-adjuvated vaccines focus on the impact on cellular immunity, and whether the elevated humoral responses elicited by STING agonists are considered effective protection is still debatable.

## 6. Application of STING Agonists in Anti-Cancer Vaccines

### 6.1. STING Agonist Vaccine Optimization

Several lethal carcinomas, such as pancreatic cancer, hepatoma, and lung cancer, exhibit an immunosuppressive microenvironment that contributes to the immune escape of cancer [72,164]. Thus, STING agonists have been introduced as promising immune-enhancing adjuvants for therapeutic cancer vaccines.

### 6.2. Melanoma

Traditional cancer treatment methods such as chemotherapy are hindered by increased drug resistance, and the STING agonist vaccine is an encouraging therapy replacement. Based on the previously described STING-LNP [165], the Nakamura group introduced a new delivery STING-LNP coated with NK cells with high-affinity synthesized cationic lipid YSK12-C4 for c-di-AMP delivery. The administration of STING-LNP combined with anti-DP-1 surprisingly generated synergic effects that stimulate NK cell activation and promote the immunosuppressive DP-1/DP-L1 response to B16-F10 melanoma lung metastasis [166]. Tumor-infiltrating lymphocytes (TILs) are essential immune cells that are involved in cancer treatment and act as biomarkers of cancer vaccine prognosis [167]. A large proportion of melanoma patients nevertheless lack TIL activation, which is an obstacle in melanoma treatment [168]. To improve therapy efficiency, Chelvanambi et al. found that intralesionally administered ADU-S100 in melanoma not only activated the tertiary lymphoid structures in the TME, providing robust secretion of anti-cancer cytokines and chemokines, but also promoted the DC-mediated inflammatory responses, increasing mouse survival rate. Importantly, the treatment also facilitated the vasculature normalization that reduced the tumor growth [169].

Researchers also investigated STING suppression in tumors. A study by Xu et al. identified trimethylation on the H3K27 enzyme, which is mediated by histone lysine methyltransferase (EZH2), which is associated with the downregulation of STING in melanoma. They then combined an EZH2 inhibitor with diABZI to increase the STING expression in melanoma and successfully reduce the tumor growth by eliciting numerous antitumor immune responses [170]. This indicates that counteracting the STING suppression pathway in cancer cells is a viable strategy to increase vaccine efficacy.

A variety of studies have also focused on combining the administration of STING agonists and the agonists of other PPRs. Temizoz et al. co-activated the TLR9 and STING signaling pathways via the synergistic effects of their agonists K3CpG and cGAMP, and the immunization showed significant antitumor effects against B16-F10 melanoma and EG-7 lymphoma and maintained a 100% survival rate for mice with lethal pancreatic cancer [171,172]. Hu et al. conjugated two artificial ligands, the STING agonist CDGSF and TLR1/2 agonist Pam3CSK4, named Pam3CSK4-CDGSF. The conjugated chemicals combined with OVA as an antigen led to a robust CD8+ response and remarkably increased the survival rate of melanoma-bearing mice [173]. CDGSF was then conjugated with another agonist, 522, for TLR7/8 by Zhang et al. In their study, CDGSF with 522 and model antigen OVA were encapsulated in NPs to enhance the immunization efficiency. Mice inoculated with melanoma attained 100% survival after the NP treatment [174]. Mice vaccinated with ADU-S100 combined with the KISIMATM, a protein delivery platform that contains a TLR2/4 agonist Anaxa, showed delayed B16-OVA melanoma and TC-1 lung tumor model development and increased survival rate. This combination exerted robust CD4+ and CD8+ T-cell responses and has proven vaccine safety [175].

### 6.3. Cervical Cancer

Existing protein-based therapeutic HPV vaccines comprise the combination of HPV E7 oncoprotein and a heat shock protein 65 (HSP65) from *mycobacterium bovis.* These vaccines are considered the most advanced vaccines against HPV but still lack immunogenicity [176,177]. The co-administration of 2′3’-cGAMP, a STING activator, with synthetic CpG-modified oligodeoxynucleotides (CpG-ODNs) and mutated HPV E7 oncoprotein is capable of activating both humoral and cellular immune response via targeting APCs and suppressing the proliferation of cervical cancer in mice [177]. To further improve the agonist delivery efficiency, Su et al. developed a nanovaccine encapsulating 2’3’-cGAMP and TC-1 cell neoantigen peptide. The researchers developed the vaccine with a pH-responsive compound, which undergoes protonation upon being engulfed into acidic cytosol, followed by the secretion of the agonist and neoantigen. In an in vivo study, the nanovaccine presented antigens on the tumor cells for a sustained period and revived the antitumor immunity in the TME [178].

### 6.4. Breast Cancer

In addition to immunotherapy drug resistance, low-immunogenicity antigens of antitumor vaccines may escape the immune system. Breast cancer vaccine development is significantly hindered by the requirement for efficient tumor-specific antigens (TAAs) [179]. To enhance the vaccine-induced immune response, Chen et al. synthesized an in situ vaccine which contains no tumor-specific antigen but only vaccine adjuvant. Adjuvant c-di-AMP was coated with poly(ethylene glycol) and ammonium-based cationic molecule modified MSNs. After intratumoral injection to the T41 breast tumor model, this in situ vaccine induced tumor cell death and the release of tumor self-antigen. Coupling with the cellular responses triggered by the agonists, the vaccine demonstrated strong antitumor effects. Their study revealed a novel design of high-immune-stimulation vaccines [180]. It is also possible to enhance antigen-induced immunity by formulating the antigen with the adjuvant. Kang et al. investigated the synergetic effect of antigen HSP90 and a STING agonist. The co-administration of HSP90 and the agonist led to an augmented HSP90-specific CD8+ response and significantly prolonged the survival time of breast-cancer-bearing mice compared to the mice that received monotherapy of HSP90 and the agonist [181].

The antigens cross-presented by APCs mainly form long-lived proteins; however, the TAAs cross-presented in tumor cells only exist in short-lived protein forms [182,183]. Increasing the exposure of short-lived presentation antigens on tumor cells is a feasible strategy to enhance a vaccine-induced immune response. As breast cancer ubiquitinated proteins (UPs) are considered potent antigens and have been characterized as short-lived proteins, Huang et al. delivered a DMXAA adjuvant UP-enriched therapeutic vaccine to 4T1/EPB breast cancer cells and elicited a robust CD8+ T-cell response. 4T1/EPB mice injected with this combination exhibited a noticeable increase in survival rate [184,185].

Taken together, the use of STING agonists as adjuvants for cancer vaccines can trigger remarkably enhanced cancer-specific cellular responses and provide a wide range of protection.

## 7. STING Agonists Are Safe as Vaccine Adjuvants

A great number of studies have demonstrated cases of inflammatory diseases derived from abnormal STING function [186], and several adverse effects of STING agonists have also been reported in pre-clinical studies (Table 2), which may lead to safety concerns on whether overexpression of STING causes undesirable immunogenic effects. Importantly, Liu et al. previously reported that the STING protein was immediately degraded after the activation of TBK1 [43]. This conclusion is consistent with the results from Prabakaran et al., who explained that TBK1 phosphorylates a selective autophagy receptor, p62. This process increases the affinity of p62 ubiquitin binding, while STING is ubiquitinated by K63-linked ubiquitin, ultimately binding to p62 for degradation [187]. Li et al. further confirmed that the recycling of STING is completed via autophagy [188]. The STING-TBK1-p62 degradation pathway enables the suspension of STING-derived autophagy and abolishes downstream immune responses to avoid the undesirable destruction of cytosolic components. Moreover, STING activity is rapidly deactivated during the transition between the ER and the Golgi and consequently degraded by lysosomes. Gonugunta et al. revealed that this process is associated with ER exit [189], and their study claimed that suspended lysosome-mediated STING degradation promotes antitumor effects. The STING degradation pathway emphasizes the safety of using STING agonists as vaccine adjuvants, sufficiently preventing systemic inflammation due to STING overexpression.

However, despite STING being depleted immediately after signaling activation, vaccine adjuvant CDN-type agonists can be easily diffused from the injection site, decreasing the efficiency of the vaccine and potentially leading to systemic inflammation. Thus, they require encapsulation with additional hydrophobic delivery systems. Hernandez-Franco et al. discovered that coating ADU-S100 with phytoglycogen NPs named Nano-11 triggers both IgG and IgA secretion against influenza A infections in pigs. It was also revealed that the antitumor drug ADU-S100 possesses antiviral effects [192,193]. On the other hand, Ebensen et al. asserted that c-di-AMP NP vaccines designed in dry powder form allow for the development of vaccines applied via inhalation to avoid unintended biological activities and enable the direct delivery of drugs into the respiratory tract. In their study, c-di-AMP was co-administered with antigen OVA NPs, and immunized mice demonstrated strong T-cell responses, indicating that this formula may play a vital role in eliminating respiratory infectious pathogens [194]. Moreover, this dry powder vaccine exhibits a high dose-sparing potential and was suggested to also be beneficial for therapeutic cancer vaccines which require the avoidance of cytotoxicity [194]. Although the abnormal bioactivity of STING might generate unintended inflammation diseases, delivering STING agonists using biocompatible vectors can resolve these problems. However, Messaoud-Nacer et al. found that inhalation of the STING agonist diABZI might lead to acute respiratory distress syndrome [81]. Relieving acute lung inflammation triggered by inhaling a STING agonist can be considered a future research focus to ensure the safety of STING agonist vaccines.

Moreover, STING agonist vaccines might be inappropriate for individuals with high STING sensitivity. Studies have found that naturally occurring gain-of-function mutations in the STING encoding gene TMEM173 lead to autoinflammatory disease [195,196]. Administrating STING agonists in individuals with this mutation might worsen the inflammatory symptoms.

## 8. Conclusions

To conclude, the STING signaling pathway is widely expressed throughout various cell types, and combining STING agonists with pathogen/cancer cell-specific antigens can provide significant adjuvant effects, including enhanced immune responses based on cytokine secretion and the activation of numerous immune cells (e.g., T cells, macrophages, DCs, and NKs). In addition to cellular responses, several studies have demonstrated that STING agonist adjuvant vaccines induced superior immune response to vaccines that were adjuvanted with other compounds. Therefore, STING agonists have high potential to be used as adjuvants in preventive and therapeutic vaccines, in order to eliminate infections and cancer cells. However, some STING agonists are easily degraded after injection or present substance toxicity; thus, the delivery of these agonists requires specific delivery platforms such as LNPs.

## Figures and Tables

**Table 2:** Side effects of STING agonists observed in pre-clinical vaccine studies.

Treatment	Route	Side Effects	Reference
2′3′-c-di-AM (PS) 2 (Rp, Rp)	IP	Delayed relapse and ascites	[190]
DMXAA	IP	Mouse body weight loss	[155]
	IP	Mouse body weight loss and attenuated motor function	[153]
	IP	Site-specific hemorrhagic necrosis	[191]
diABZI	ET	Acute respiratory distress syndrome	[81]
Soluble cdGMP	SC	Systemic inflammatory toxicity	[148]

IP, intraperitoneal administration; ET, endotracheal administration; SC, subcutaneous administration.

## Data Availability

The study did not report any data.

## Abbreviations

The following abbreviations are used in this manuscript:

STING	Stimulator of interferon genes
hSTING	Human-encoded STING
PRR	Pattern recognition receptor
PAMP	Pathogen-associated molecular pattern
dsDNA	Double-strand DNA
CDN	Cyclic dinucleotide
cGAS	GMP-AMP synthase
ERGIC	ER–Golgi intermediate compartment
TBK1	Tank-binding kinase 1
IRF3	Interferon regulatory factor 3
IFN	Interferon
ISG	IFN-stimulated gene
NK	Natural killer
CIN	Chromosomal instability
DC	Dendritic cell
eDNA	Extracellular DNA
pDNA	Plasmid DNA
ESAT-6	Early secretory antigenic target
siRNA	Small interfering RNA
NP	Nanoparticle
DMXAA	5,6-Dimethylxanthenone-4-acetic acid
ABZI	Amidobenzimidazole
LNP	Liposomal nanoparticle
MSN	Mesoporous silica nanoparticle
Mtb	*Mycobacterium tuberculosis*
TME	Tumor microenvironment
Ace-DEX	Acetalated dextran
OMV	Outer membrane vesicle
DacA	Diadenylate cyclase
ThyA	Thymidylate synthase
NE	Neutrophil
HA-Mal	Hyaluronic acid-maleimide
BCG	Bacille Calmette–Guérin
CDG	Cyclic-dyguanylate
SAL	STING-agonist-derived amino lipid
DOPE	2-dioleoyl-sn-glycero-3-phosphoethanolamine
RBD	Receptor binding domain
GP	β-glucan particle
nAb	Neutralization antibody
HSE	Herpes simplex encephalitis
HSV	Herpes simplex virus
HBV	Hepatitis B
TLR	Toll-like receptor
TIL	Tumor-infiltrating lymphocyte
EZH2	Histone lysine methyltransferase
TAA	Tumor-specific antigen
UP	Ubiquitinated protein
